# Feline sacroiliac luxation: comparison of fluoroscopy-controlled freehand vs. computer-navigated drilling in the sacrum—a cadaveric study

**DOI:** 10.3389/fvets.2024.1510253

**Published:** 2025-01-15

**Authors:** Lukas Kleiner, Nicole Wolf, Christina Precht, Kati Haenssgen, Franck Forterre, Pia Düver

**Affiliations:** ^1^Divison of Small Animal Surgery, Department of Clinical Veterinary Medicine, Tierklinik Marigin, Feusisberg, Switzerland; ^2^Divison of Small Animal Surgery, Department of Clinical Veterinary Medicine, Vetsuisse-Faculty, University of Bern, Bern, Switzerland; ^3^Divison of Clinical Radiology, Department of Clinical Veterinary Medicine, Vetsuisse-Faculty, University of Bern, Bern, Switzerland; ^4^Divison of Veterinary Anatomy, Department of Clinical Research and Veterinary Public Health, Vetsuisse-Faculty, University of Bern, Bern, Switzerland

**Keywords:** neuronavigation, stealth station S8, computer-navigated surgery, sacroiliac luxation, cat, cadaveric study

## Abstract

**Introduction:**

Sacroiliac luxation is a common traumatic feline injury, with the small size of the sacral body being a challenge for surgical stabilization. This study compared an innovative computer-guided drilling method with the conventional fluoroscopy-controlled freehand technique. Neuronavigation, using CT-based planning and real-time tracking, was evaluated against the freehand method for accuracy and time efficiency.

**Materials and methods:**

Bilateral sacroiliac luxation was induced in 20 feline cadavers. One side of the sacral body was drilled using fluoroscopy, and the other with neuronavigation (Stealth Station S8). A reference frame was affixed to the sacral spinous process for tracking. Ten cats were operated on by an ECVS diplomate and 10 by a resident. Postoperative cone beam CT images were used to assess both techniques, comparing the accuracy of the planned corridor vs. the actual drill hole in the sacrum. A learning curve for both methods was estimated by measuring procedure time.

**Results:**

CT scan assessments showed all 40 drill holes achieved “surgically satisfactory” results. The computer-navigated technique demonstrated an average deviation of 1.9 mm (SD 1.0 mm) at the entry point and 1.6 mm (SD 0.8 mm) at the exit point. The pins of 3/20 reference frames penetrated the vertebral canal, creating a risk for potential clinical complications. The neuronavigation-guided procedures took an average of 23 min and 37 s (SD 8 min 34 s), significantly longer than the freehand technique, which averaged 9 min and 47 s (SD 3 min 26 s). A steep learning curve was observed with neuronavigation.

**Discussion:**

The neuronavigation-guided technique achieved accuracy is comparable to the fluoroscopy-controlled method, is offering real-time feedback and has potential for highly precise surgeries near critical anatomical structures. However, significant attention must be given to the placement of the reference frame, as it is placed blindly and presents a potential risk for errors and complications. Despite its longer duration, the neuronavigation method shows promise for improving precision in complex surgical scenarios.

## 1 Introduction

One of the most common fracture sites after trauma in cats is the pelvic region (1). Up to 60% of cases of pelvic fractures involve the sacroiliac joint. Sacroiliac fracture luxations are injuries most commonly observed following falls from height or after motor vehicle trauma and usually occur in combination with other pelvic fractures or soft tissue injuries. In cats, sacroiliac dislocations are bilateral in approximately 15% of cases (1).

Based on the radiologically assessed grade of displacement and the clinical symptoms, either conservative management or surgical treatment may be considered. Surgical stabilization is indicated in cases of severe pain, neurologic deficits, pelvic canal stenosis, and/or when rapid return to weight-bearing activity is desired (2–4). The most common surgical method is the placement of a lag screw through the iliac wing into the sacrum, either through an open or minimally invasive approach. Both methods are usually performed under fluoroscopic control, but can also be performed without fluoroscopy (5–7). There are also reports of successful stabilization using single transiliosacral pin (8), transiliosacral toggle suture (9), transileal pin/bolt/screw (10), ventral screw placement (11) and tension band technique (12). Placement of the lag screw within the sacral body is the most critical part of the procedure, as the safe corridor is small and there are several important structures which should not be damaged during the surgery (termination of the spinal cord and nerve roots dorsally, intervertebral disc cranially and important nerves and vascular structures ventrally). The optimal entry point and angle of the screw were analyzed in several studies (13–15). For sufficient bone purchase, the screw should penetrate 60% of the sacral diameter (16).

Previous studies evaluated open and minimally invasive placement of lag screws under fluoroscopic control. Malpositioning of the screw was seen in 7.5–12.5% of cases, which directly led to revision surgery or had an increased risk of postoperative implant loosening (5, 7, 10, 16).

In human medicine, computer technology has been used in the operating room for years to improve the accuracy and safety of various procedures. Especially for brain and spine surgery, neuronavigation enables accurate implant placement by visualizing the corridor being drilled and any deviations of the planned corridor in real-time (17–19). The technique is considered safe and effective in humans for sacroiliac joint surgery (20–22).

In veterinary medicine the use of neuronavigation is described for placing toggle constructs across the coxofemoral joint and fracture treatment in horses (23–25), minimally invasive spinal stabilization in dogs (26), vertebral pin placement in dogs (27), craniectomies in dogs with osteochondrosarcoma (28) and brain biopsies in dogs and cats (29). In horses this technique is used in clinical cases to place implants in the proximal phalanx, the third metatarsal bone, the ulna or the medial femoral condyle (24).

To date, the author is not aware of any studies on the application of neuronavigation for the surgical treatment of feline sacroiliac luxation. The purpose of this study was to evaluate the accuracy and safety of this technology for treatment of feline sacroiliac luxation. We hypothesized that drilling a hole in the sacrum using computer-navigated drilling through neuronavigation would be at least as accurate as conventional freehand drilling under fluoroscopic control. Additionally, we expect a steep learning curve, particularly for computer-navigated drilling, due to improvement in workflow execution and the application of the new technique. This is expected to affect the overall surgery duration.

## 2 Materials and methods

Bilateral sacroiliac luxations were created in 20 feline cadavers and a drill hole was placed into the sacral body on each side. One side was operated on using conventional fluoroscopy-controlled freehand technique and one side was operated on using the new neuronavigation technique.

### 2.1 Cadavers

Twenty cadavers of skeletally mature cats were included in the study. All cats died or were euthanized due to medical conditions unrelated to this study at the clinics of the authors. Cats had to be free of pelvic and sacral pathologies, which was confirmed by an initial cone beam computed tomography (CBCT; O-arm, Medtronic, Louisville, Colorado) scan. Thus, exclusion criteria were pelvic fractures, sacroiliac luxation, lumbosacral (sub)luxation, sacral fractures and neoplasia.

All cadavers were prepared in an identical manner. A ventral approach to the pelvic symphysis (30) was performed in dorsal recumbency. The pelvic symphysis was then separated using an oscillating saw (Colibri II; DePuy Synthes, West Chester, PA) to simulate the instability occurring in clinical cases due to additional pelvic fractures. Finally, a standard dorsolateral open approach to the wing of the ilium and dorsal aspect of the sacrum (30) was performed in lateral recumbency to manually separate the sacrum from the ilium with a raspatorium. This was done bilaterally resulting in a total of 40 sacroiliac luxations.

### 2.2 Order of procedures

All procedures were performed by either an ECVS Diplomate or a second year ECVS resident. The planning for the computer-navigated technique was performed by another ECVS resident, and had no prior experience with the navigation software before. The decision as to which of the two surgeons would begin with the first cadaver was randomly assigned. The two surgeons then took turns to conduct the surgery to ensure that the learning curve of the person doing the planning was distributed as evenly as possible. Both techniques [computer-navigated (CN group) vs. fluoroscopy-controlled freehand (FC group)] were performed on each cadaver. The decision as to which side of each cadaver was used for fluoroscopy-controlled and which was used for computer-navigated surgery, as well as which side was started with, was randomized.

As a result, each surgeon performed 10 procedures of each technique.

### 2.3 Technical equipment

For intraoperative imaging a mobile CBCT unit (O-arm) was used, which was connected to the surgical navigation system StealthStation S8 (Medtronic) and provided two-dimensional and 3D images. The StealthStation S8 (SS) allows tracking of the surgical instruments relative to the patient’s anatomy on a screen.

The unit was used either for fluoroscopy (FC group) or to perform a CBCT and neuronavigation (CN group).

### 2.4 Surgical procedure

All animals were placed in lateral recumbency for both procedures, controlled by fluoroscopy with the O-arm. Correct positioning (was achieved with towels) was evaluated by superimposition of the transverse processes of the seventh lumbar vertebra. The approach to the wing of the ilium and the dorsal aspect of the sacrum was already performed during cadaver preparation.

The goal of both techniques was to position the drill hole centrally within the first sacral body, with its end at 50% of the mid-transversal width.

After completion of each procedure, a control CBCT was performed to evaluate the drill hole within the sacrum.

#### 2.4.1 Computer-navigated drilling

The neuronavigation system from Medtronic consists of two parts: the O-arm for performing fluoroscopy and CBCT scans and the SS, a surgical navigation system that allows the surgeon to follow the position of instruments live on the screen during surgery. The position of the patient and the instruments is determined by optical tracking via an infrared camera. For this purpose, a reference frame with 4 reflecting spheres needs to be attached to the bone, into which the navigated hole will be drilled, while the drill used must be equipped with a tracker (sure track 2, Medtronic).

With this system, the surgeon can plan a corridor on the 3D reconstruction on the screen and track the instruments in different planes in real time during surgery.

The placement of the reference frame in the spinous process of the feline sacrum is performed without direct visualization. An anatomical study assessed the optimal position of the pin from the reference frame to minimize the risk of compromising critical anatomical structures.

##### 2.4.1.1 Anatomical landmarks for reference frame placement

**Figure 1:**
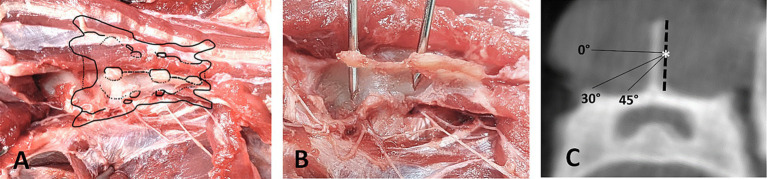
Landmarks for reference frame placement. **(A)** Dorsal view on dissected feline sacrum with visualization of the dorsal sacral foramina. **(B)** Position of accurately placed pins in the spinous processes of S1 and S2, making contact to the sacral roof medial to the dorsal sacral foramina. **(C)** Transverse CBCT-Image of S1 showing the safe angle for placing the reference frame pin, starting at the mid-height of the spinous process (*).

The skin and dorsal musculature over the sacrum, medially to the bilateral dorsolateral approach, were dissected and reflected caudally. The fascial layer and fatty tissue were subsequently removed. The dorsal sacrococcygeal muscle was then detached from the sacrum to expose the dorsal sacral foramina. With the dorsal sacral foramina exposed, the dorsal rami were carefully dissected and visualized (Figure 1A).

To achieve adequate stabilization of a smooth pin in the small feline sacrum, two fixation points are required: one at the spinous process of the first or second sacral vertebra and another at the sacral roof (Figure 1B). Ensuring a secure hold in the spinous process necessitates precise placement of the pin. Inserting the pin too dorsally in the spinous process may not provide sufficient stability, while inserting it too ventrally risks penetrating the vertebral canal. Therefore, the optimal insertion point is at the transition from the middle to the dorsal third of the spinous process. This strategic placement minimizes the risk of vertebral canal penetration while maximizing the anchoring strength necessary for effective stabilization. The pin should be placed at an angle of 30–45 degree angle (Figure 1C) to avoid entering the vertebral canal medially and the dorsal sacral foramina laterally. Especially when targeting the second spinous process, a steep enough angle needs to be chosen to avoid the dorsal sacral foramen where the dorsal branch of the second sacral nerve emerges. The dorsal branches of the sacral nerves are interconnected and innervate the lateral and medial dorsal sacrococcygeal muscles, as well as the overlying skin areas. In the event of nerve irritation or injury, the likelihood of significant complications remains low due to the limited functional significance of the affected structures (31).

##### 2.4.1.2 Reference frame placement

The cadavers were placed in the lateral position on the table and a blunt dissection was performed down to the spinous process of the first or second sacral vertebra through the existing incision from the former approach.

**Figure 2:**
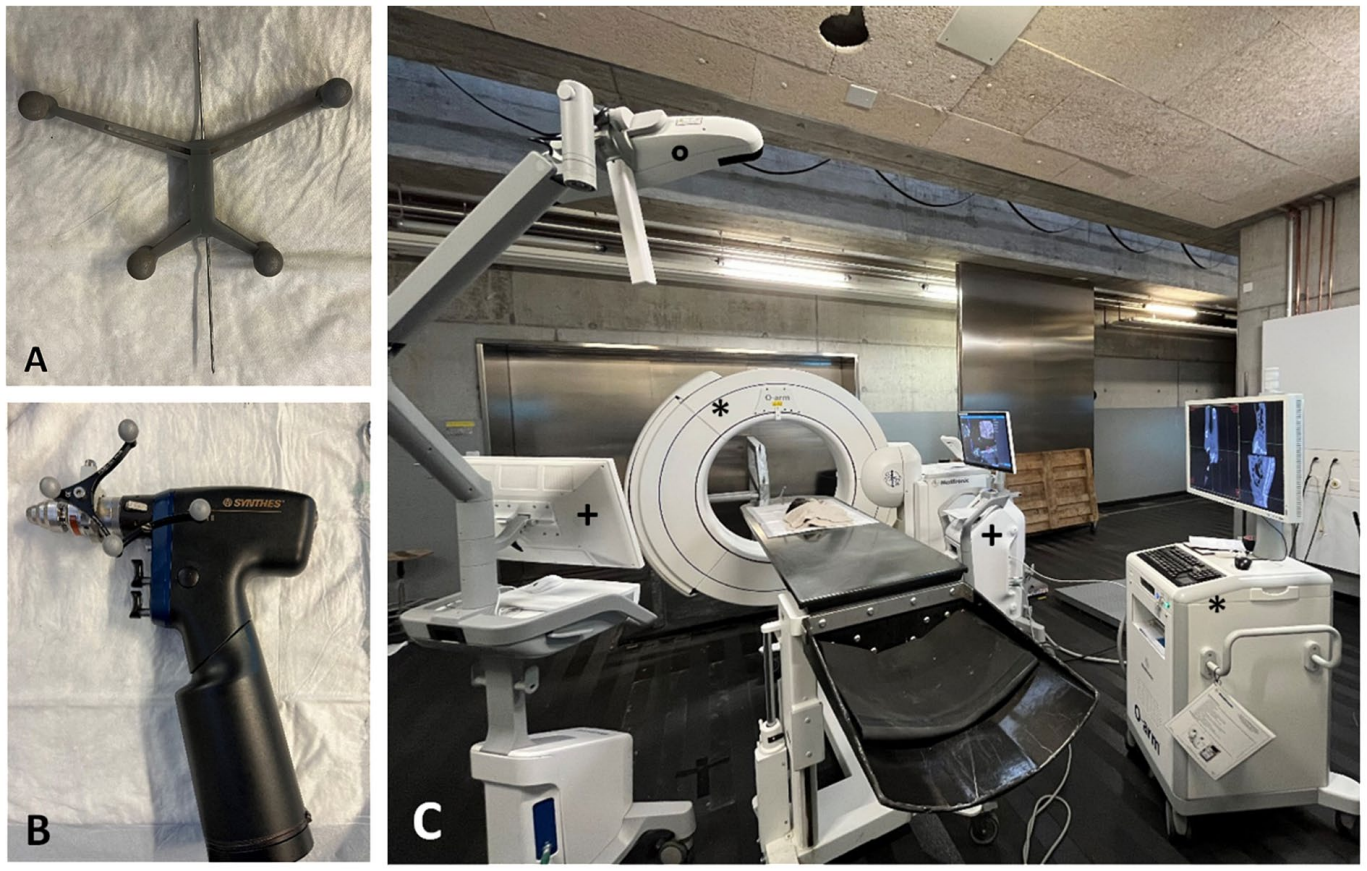
**(A)** 3D-printed reference frame with pre-positioned 1.4 mm smooth pin. **(B)** Surgical drill (Colibri II) with sure track 2 allows the surgeon to follow the position of the drill tip live on the screen. **(C)** Set-up for the computer-navigated drilling: O-arm with screen (*), Stealth Station S8 with two screens (+) and the infrared camera for optical tracking (o).

A 3D-printed reference frame (Figure 2A) was used. The material (Tough 1,500 Resind, Formlabs Inc., Somerville, United States) is light weighted and was printed with six central holes. A smooth 1.4 mm pin was drilled through one of these holes using press fit fixation. The pin with the pre-positioned reference frame was then placed using a surgical drill (Colibri II), according to the anatomical study, starting in the middle third to dorsal third of the height of the spinous process and at a 30 to 45-degree angle from ipsilateral through the spinous process of the first or second sacral vertebra in contralateral direction to anchor in the roof of the sacrum. The choice which of the two spinous processes to use was made individually, and in cases of malpositioning or fracture, the alternate spinous process was utilized.

The pin was then bent dorsally toward the table to prevent the reference frame from interfering with the drilling device and to ensure that the spheres on the reference frame could be detected by the infrared camera at all times.

The position of the reference frame pin was evaluated on the postoperative CBCT images.

##### 2.4.1.3 Computer-navigated drilling

The camera of the SS was positioned so that it was able to detect both the reference frame with at least three of the four reflecting spheres and the reflecting reference points on the O-arm simultaneously (Figure 2C).

The CBCT images are uploaded directly to the SS. The corridor is planned by defining the entry and target points on the 3D reconstruction.

**Figure 3:**
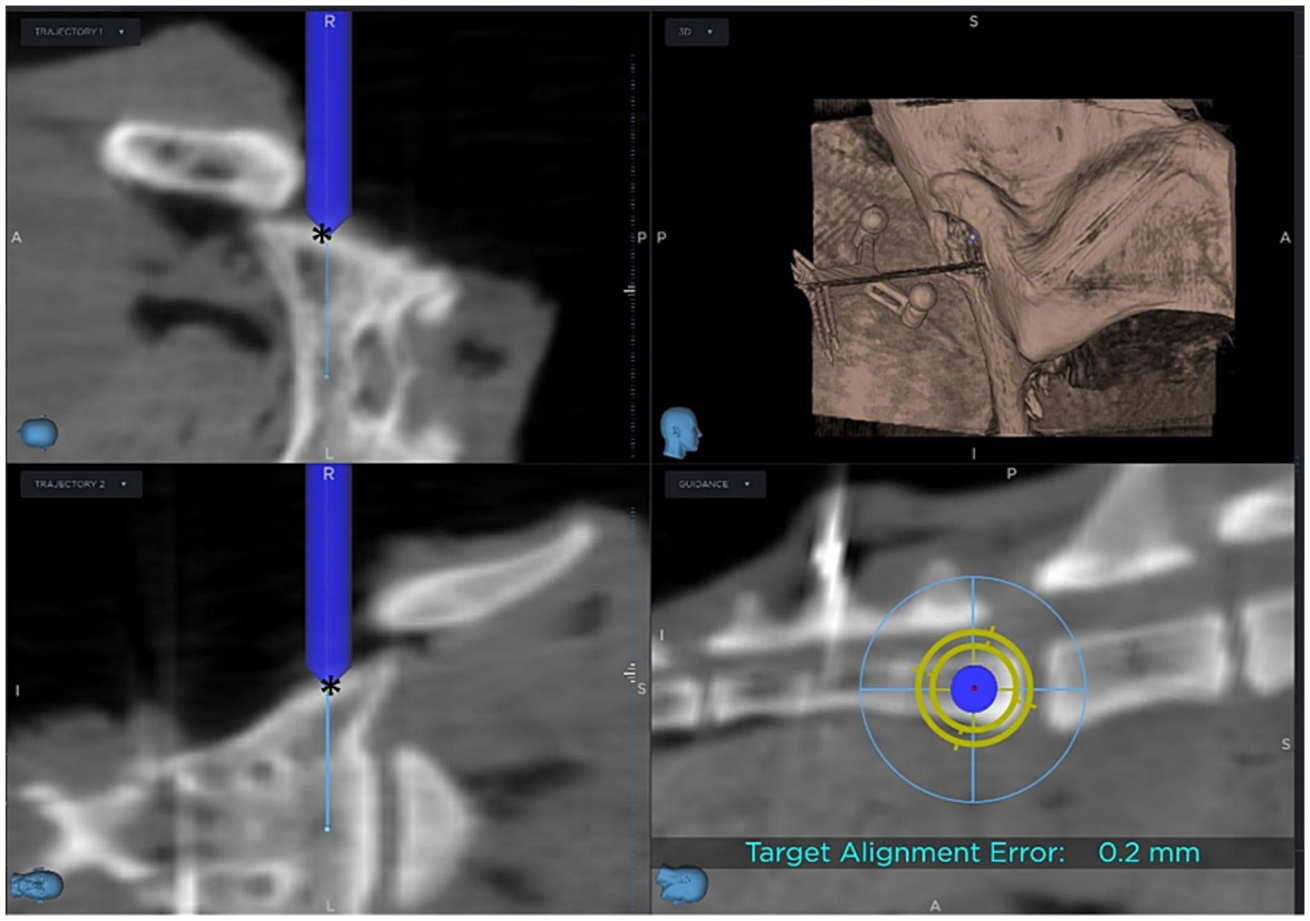
Screen view during computer-navigated drilling: The blue cylinder represents the drill bit, with its tip positioned at the entry point (*) of the planned corridor.

Before the planned corridor could be drilled, the required instruments (sure track 2 (Figure 2B) and passive planar sharp, Medtronic) had to be registered and calibrated with the tracking device of the SS. Verification of correct registration is essential during computer-navigated surgery to ensure that the position of the instrument on the patient reflects the position of the instrument on the same anatomical structure on the screen. To confirm correct calibration, the tip of the drill bit was positioned adjacent to the entry point of the pin of the reference frame on the spinous process and it was accepted only if the position could be verified in all three planes on the screen. If the images on the screen showed an identical position as the drill bit on the patient, drilling into the sacral body along the planned corridor could be started. The drill hole is then drilled using the guidance on the screen (Figure 3). In the initial two cases, a 1.8 mm drill was utilized. Due to the presence of a slightly inclined joint surface, which increased the risk of drill slipping, the initial drill size was changed in the following cases to 1.1 mm. The 1.1 mm drill hole was over drilled with a 1.8 mm drill bit.

#### 2.4.2 Fluoroscopy-controlled drilling

The iliac wing is retracted by an assistant, to allow visualization of the sacral wing. The surgeon placed a 0.8 mm Kirschner wire in the sacral wing using a surgical drill and aimed for the described position for a drill hole in the sacral body of cats (14, 16). The position of the wire was controlled under fluoroscopy and corrected if necessary. Targeting was focused on the center of the first sacral body. Depending on the surgeon’s preference a 0.8 mm Kirschner wire was used for all attempts or the size was changed to a 1.25 mm Kirschner wire. Fluoroscopic control was performed after each repositioning. Afterwards the hole within the sacral wing and body was drilled with a surgical drill bit. Depending on surgeons’ preference the determined position was first drilled with a smaller drill bit (1.1 mm, 1.5 mm) and then finalized with a 1.8 mm drill bit or no predrilling was performed.

### 2.5 Evaluation of the accuracy of the computer-navigated procedure

To assess the accuracy of the computer-navigated technique, entry and target points for the drilled holes were defined on postoperative CT images using SS software (Medtronic), analogous to the initial plan. Pre- and postoperative CT images were manually superimposed using the SS software. This allowed a direct comparison between the initial planned corridor and the actual drill hole. To objectify the assessment, the coordinates of the respective entry and target points were described by the following distance formula (26):


$$\mathrm{Deviation}\;\left[\mathrm{mm}\right]=\sqrt{{\left(X2-X1\right)}^{2}+{\left(Y2-Y1\right)}^{2}+{\left(Z2-Z1\right)}^{2}}$$

### 2.6 Radiologic evaluation and comparison of the two techniques

The postoperative CBCT images were evaluated by a Diplomate of the European College of Veterinary Diagnostic Imaging (DipECVDI). The assessment focused on the positioning of the drill holes in all 40 cases and the positioning of the reference frame pins in the 20 cases of the SN group.

Drill holes were classified using a grading scale (Table 1) into Grades 1 to 3. Grade 1 indicated optimal placement within the vertebral body, Grade 2 denoted drill holes in contact with or eroding the cortex, and Grade 3 signified cortex violation. Grade 1 and 2 can be considered satisfactory operation results. Grade 3 is regarded as unsatisfactory surgical result because violation of surrounding structures may occur. For Grades 2 and 3, the direction of the deviation was further described using letters a-d, describing the direction of deviation (a = deviation in dorsal direction; b = deviation in ventral direction; c = deviation in cranial direction; d = deviation in caudal direction).

**Table 1:** Safety drilling coridors.

Safety grade	Safety name	Description
1	Well IN	Trajectory within the vertebra, intact cortex of the vertebral canal floor and the ventral aspect of the sacrum
2a	Just, IN	Trajectory within the vertebra, in contact or eroding the cortex of the vertebral canal floor
3a	Too Far, IN	Trajectory violating the cortex of the vertebral canal floor or within the vertebra canal
2b	Just, Out	Trajectory within the vertebra, in contact or eroding the ventral aspect of the sacrum
3b	Too Far, Out	Trajectory violating the ventral cortex of the sacrum or ventrally outside of the sacrum
2c	Just, CRAN	Trajectory within the vertebra, in contact or eroding the cranial endplate of S1
3c	Too Far, CRAN	Trajectory violating the cranial endplate of SI or within the IVDS L7/S1
2d	Just, CAUD	Trajectory within the vertebra, in contact or eroding the caudal endplate of S1
3d	Too Far, CAUD	Trajectory violating the caudal endplate of SI or within S2

Classification scheme to assess safety. The drilling corridor is assessed in the 60% ipsilateral part of the vertebral body and wings of S1.

Grades 1 and 2 were categorized as “surgically satisfactory” results since adequate stability is ensured despite cortical erosion. Additionally, this classification is supported by the fact that no significant anatomical structures are compromised or injured.

The position of the reference frame pins was evaluated using a modified grading scale (Table 2). Grade 1 indicated optimal placement through the spinous process into the sacral wing, while Grade 3a represented penetration into the spinal canal, and Grade 3b indicated penetration of the ventral cortex.

**Table 2:** Safety patient tracker.

Safety grade	Safety name	Description
1	IN	Trajectory within the dorsal spinous process of S1 or S2 and unilaterally in the sacral wing of S1 or S2
3a	Too Far, IN	Trajectory within vertebral canal
3b	Too Far, OUT	Trajectory in soft tissues ventrally to the sacrum

Modified classification scheme to assess safety of the patient tracker.

### 2.7 Evaluation of surgical time

Time was recorded for all procedures. As the approach to luxate the sacroiliac joint, was already performed in all cases “Time total” started with manipulation of the luxated sacroiliac joint by the surgeon/assistant to place the first Kirschner wire in the FC-group and with placing the reference frame in the CN-group. It ended in both groups with finishing the 1.8 mm drill hole within the sacral body. The “time total” was divided into “time on patient” and “time off patient.” “Time on patient” was defined as time needed for the surgical procedures itself (manipulation to visualize the sacral body, placement of reference frame or Kirschner wires and the drilling process itself). “Time off patient” was defined as the time required for all the imaging procedures (both groups) and planning of the drill hole (CN-group).

### 2.8 Statistical analysis

To assess the effect of the surgery technique on the total surgery time and the two partial times “time off patient” and “time on patient,” a Wilcoxon signed-rank test was used after demonstrating the violation of the normality distribution using the Shapiro–Wilk test of normality. Additionally, visual inspection showed a right-skewed distribution of the data and a nonlinear distribution of the residues in the quantile-quantile plot. As the two surgeries were performed on each side of the same cadaver, the surgeries were considered as paired samples to account for an eventual effect of the cadaver on the outcome. To compare the effect of the two surgeons an unpaired sample Wilcoxon was used instead. A post-hoc power calculation was performed and confirmed that the sample size n = 20 for each technique was sufficient to reach a power of 96%.

To understand the learning effect, the first 10 surgeries (first five of each surgeon) were grouped and compared to the last 10 using the unpaired sample Wilcoxon signed-rank test. The data splitting in two groups reduced the previously described skewness of the data distribution. However, as the sample size for each group was also reduced to *n* = 10 we decided to use a non-parametric test, as a normal distribution is difficult to prove with a small sample size.

To compare the frequencies of the gradings assigned to the drill holes for the radiologic evaluation, the Fisher’s exact test was chosen as for some of the grades there were less than five observations.

The accuracy of the guided procedure deviation to the target at the “entry” and “target” position was compared between the two surgeons using the t-test, as the data points suggested normal distribution in the Q-Q plot visualization, and this impression was confirmed with a Shapiro–Wilk normality test (*n* = 10 in each group).

The statistical analysis was performed using the statistical software R (version 4.3.1) in RStudio (Posit Software, version 2023.09.1.494).

## 3 Results

### 3.1 Study population

The cadavers of 20 skeletally mature cats (16 domestic shorthair, one domestic longhair, one Birman, one Persian, one Siamese) were included, of which 13 were males and seven were females with weights ranging from 1.78 to 5.49 kg (mean 3.45 kg).

### 3.2 Evaluation of the accuracy of the computer-navigated procedure

#### 3.2.1 Neuronavigation

Across all 20 cases, the average deviation was 1.9 mm at the entry point (standard deviation (SD) 1.0 mm), and 1.6 mm at the target point (SD 0.8 mm). The experienced surgeon demonstrated a mean deviation of 1.9 mm at the entry point (SD 0.5 mm) and 1.8 mm at the target point (SD 0.9 mm). In contrast, the inexperienced surgeon demonstrated a mean deviation of 2.0 mm at the entry point (SD 1.4 mm) and 1.5 mm at the target point (SD 0.6 mm).

There was no statistically significant difference in drill hole accuracy between the two surgeons. When comparing the first four cases to the last four cases no statistically significant difference was observed.

### 3.3 CT scan evaluation of the drill holes

#### 3.3.1 Neuronavigation

**Figure 4:**
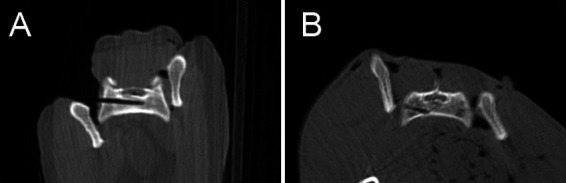
Postoperative CBCT-images showing a drill hole judged as grade 1 “Well IN” **(A)** and another one as grade 2b “Just OUT” **(B)**.

In the CN group, 11/20 cases were judged as Grade 1 (Figure 4A), with 2 /20 cases classified as Grade 2a and 7/20 cases as Grade 2b (Figure 4B). The experienced surgeon achieved Grade 1 in 5/10 cases, Grade 2a in 1/10 cases, and Grade 2b in 4/10 cases. The inexperienced surgeon achieved Grade 1 in 6/10 cases, Grade 2a in 1/10 cases, and Grade 2b in 3/10 cases.

All 20 cases achieved “surgically satisfactory” results.

No statistically significant difference concerning the grading was found between the surgeons.

#### 3.3.2 Fluoroscopy

In the FC group, 15/20 cases were classified as Grade 1, and 5/20 were Grade 2b. With the experienced surgeon achieving 8/10 Grade 1 and 2/10 Grade 2b, while the inexperienced surgeon reached 7/10 Grade 1 and 3/10 Grade 2b.

All 20 cases achieved “surgically satisfactory” results.

#### 3.3.3 Comparison of the two techniques

All drill holes from both groups were either centrally located within the vertebral body (Grade 1) or in contact with the vertebral cortex (Grade 2). None of the drill holes breached the cortex (Grade 3), indicating satisfactory surgical outcomes for all cases.

Furthermore, neither in the FC-group nor in the CN-group, did any of the drill holes extended into the proximity of the cranial vertebral endplate or caudal vertebral border. No statistically significant difference was found between the two techniques concerning the radiological evaluation of safe drill corridors in the sacral body.

### 3.4 CT scan evaluation of the reference frame

#### 3.4.1 Neuronavigation

In 16 out of 20 cases, the pin was satisfactorily anchored in the 1st or 2nd spinous process and sacral wing. In one case (case 1) from the experienced surgeon, the pin extended through the sacral wing into the soft tissue ventrolateral to the sacral wing. In 3 cases (cases 2, 3, 10), one from the experienced surgeon and two from the inexperienced surgeon, the pin extended into the spinal canal.

In 6 out of 20 cases, the reference frame had to be placed twice: in three cases (cases 2, 13, 14) due to spinous process fracture, in one case (case 3) due to a blunt pin, in one case (case 18) due to poor recognition by the camera, and in one case (case 17) due to insufficient pin anchoring resulting in unstable fixation.

### 3.5 Surgery time

#### 3.5.1 Neuronavigation

For all 20 cases, the average duration was 23 min 37 s (SD 8 min 34 s). The average “time off patient” was 12 min 17 s (SD 5 min 11 s), while the average “time on patient” was 11 min 19 s (SD 6 min 17 s). 60% of the time reduction was achieved during the ‘time off patient’ phase, while the remaining 40% was saved during the ‘time on patient’ phase.

**Figure 5:**
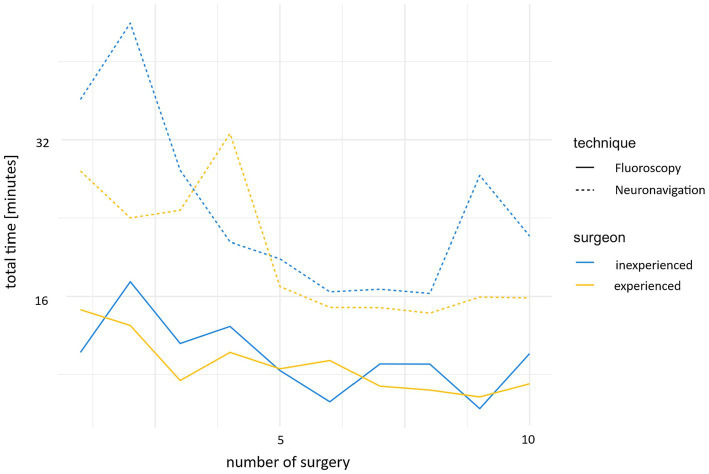
Development of the time required for neuronavigation and fluoroscopy during the study, comparing the experienced and inexperienced surgeons.

The surgery time was significantly reduced after a few cases (*p* < 0.001). While the first 10 cases took an average of 28 min 55 s (SD 8 min 29 s), the time was reduced to 18 min 19 s (SD 4 min 33 s) for the second 10 cases (Figure 5).

The experienced surgeon took an average of 5 min less than the inexperienced surgeon for both the first and second 10 cases; however, no statistically significant difference between the surgeons was found.

#### 3.5.2 Fluoroscopy

For all 20 cases a mean duration of 9 min 47 s (SD 3 min 26 s) was noted. The “time off patient” took 3 min 54 s in average (SD 1 min 42 s), while the “time on patient” lasted 5 min 52 s (SD 2 min 26 s).

An improvement in time was also observed in the fluoroscopically controlled cases. While the first 10 cases took an average 11 min 54 s (SD 3 min 16 s), this time was reduced to 7 min 39 s (SD 2 min 3 s) for the second 10 cases (Figure 5).

A non-statistically significant difference of approximately 1 min was noted in the mean “time total” between the experienced and the inexperienced surgeon.

#### 3.5.3 Comparison of the two techniques

The CN group exhibited a markedly longer procedural duration compared to the FC group, with this difference being statistically significant (*p* < 0.001).

## 4 Discussion

Our initial hypothesis regarding the safety of neuronavigation is partially supported, given that there was no statistically significant difference observed between the computer-navigated and fluoroscopy-controlled freehand surgical techniques in terms of sacral drilling. However, the placement of the reference pin in the spinous process posed an increased risk of penetrating critical structures. Furthermore, we can accept our second hypothesis as surgery time decreases significantly with experience.

All drilled holes in both techniques resulted in surgically satisfactory outcomes. Both methods demonstrated a good performance compared to a previous study on lag screw stabilization of cadaveric feline sacroiliac luxation, with an error rate of 8% and clinical studies including dogs and cats with complication rates up to 12.5% (5, 7, 16).

The high accuracy of neuronavigation is reflected in the small deviation between the planned and drilled entry and target points, with 1.9 mm (SD 1.0 mm) and 1.6 mm (SD 0.8 mm). A comparable study evaluated the accuracy of minimally invasive drilling screw corridors in the thoracolumbar spine and found slightly larger discrepancies between the planned and actual drill hole positions, with a significant difference between an experienced (mean deviation for the entry point of 2.2 mm and for the target point of 3.0 mm) and a novice surgeon (mean deviation for the entry point of 3.7 mm and for the target point of 5.0 mm) (26). It should be noted that this involved a different anatomical location, was conducted using a minimally invasive approach and drilling was performed by a 2.5 mm drill bit, which limits the direct comparability of the results.

During the present study it was noticed that accurate drilling with neuronavigation was simplified by predrilling with a smaller drill bit (1.1 mm). It reduces the risk of slippage on the slightly oblique surface of the sacral wing during the initial rotation of the drill bit. As well the drill bit is shorter and therefore less prone to bending, what was found to introduce discrepancies between the actual position of the drill tip and the calculated position based on the sensor within the handpiece. In human medicine, longer drill bits are associated with increased inaccuracies, particularly when used with small diameter drill bits. To address this issue, specialized alignment devices have been developed to enhance accuracy and mitigate potential errors during drilling procedures (32). The weight of the Colibri handpiece was another factor found to make accurate drilling more difficult in the present study. A pencil-shaped drill device could potentially simplify the drilling process at this anatomical location.

Regarding safety, it is noteworthy that the challenges encountered in positioning the reference frame, particularly at the beginning of the study, somewhat diminish the perceived safety of neuronavigation. Placing the reference frame emerges as the most critical step of the computer-navigated drilling. Ensuring the correct and stable placement of the reference frame is crucial for the successful execution of a computer-navigated procedure. Any displacement of the reference frame could lead to navigation inaccuracies and compromise the surgical outcome. Therefore, meticulous attention to the positioning and stability of the reference frame is essential to ensure accurate navigation during the procedure. In two cases in this study, movement of the reference frame resulted in a discrepancy between CBCT images and patient positioning. This was noticed during preoperative checks, and the reference frame was repositioned, allowing the surgery to proceed without complications. This highlights the importance of preoperative verification of the correlation between the position of the instruments displayed on the screen and their actual position in the surgical field.

Spinous process fracture occurred in four cases, attributed to excessively dorsal pin placement, as the tip of the process lacks adequate stability due to very thin bone. Even no clinical relevance is expected from minor fracture at the tip of the spinous process, correct positioning is essential to reduce possible complications.

In four cases the positioning of the reference frame pin was incorrect. One pin traversed from the sacral wing slightly into the ventrolateral soft tissue, which is not expected to cause any clinical complications. Three pins penetrated the cortex of the vertebral canal. The termination point of the spinal cord in cats remains controversial in the literature, with reports varying between the caudal end of L7 and S3. This variability may be influenced by factors such as age and breed-specific differences, though these remain under discussion (4, 31, 33). The clinical significance of penetrating the vertebral canal at this level remains uncertain, therefore it should be avoided to prevent potential complications. With practice, the risk of penetrating the spinal canal might be reduced, as can be seen from the fact that the experienced surgeon no longer had any complications with the pin after the third case.

To reduce the risk of penetrating vital structures while positioning the pin of the reference frame, various techniques could be further evaluated. An alternative to smooth pins would be the utilization of threaded pins, which are proven to result in more stable constructs (34) and therefore could be anchored into the spinous process only, avoiding the risk of penetrating the spinal canal. In several studies from human and veterinary medicine fixation of the reference frame on the spinous process with a clamp is described (19, 26). In the authors opinion this technique is not feasible in the cat due to the small size of the sacral spinous processes. In a different study from human medicine with unilateral affected sacroiliac joint, anchoring the reference frame into the ilium on the non-affected site is described (20). Another described technique connects the reference frame to the operating table or a device fixated to the patient, respectively, the bone of interest (19, 24). However, the bone must be rigidly fixed, as even the slightest movement may jeopardize the success of the procedure. In the authors opinion stable fixation of the small and light weighted feline sacrum is difficult but could be investigated in further studies.

Safety extends beyond the drilling process itself and heavily relies on the experience of the surgical team, especially in procedures involving neuronavigation. Proper operation of equipment and the effective use of software require coordinated efforts and familiarity among team members. Ensuring that each member is well-versed in their specific role can significantly enhance the efficiency and accuracy of the procedure. Thus, training and practice are essential to optimize outcomes and reduce time when using advanced navigation techniques.

Time is a crucial factor in surgery, as prolonged anesthesia not only places additional stress on the patient but also increases the risk of complications (35). Despite a significant time-reduction noted over 20 cases in the CN-group, the average duration for procedures using neuronavigation remained longer compared to the fluoroscopy-controlled freehand procedures. Especially, the “time off patient,” was found to be longer for the CN-group. These findings suggest that although neuronavigation enables accurate surgical planning and procedures, its implementation requires additional time for preoperative planning compared to fluoroscopy. To minimize the duration as effectively as possible, it is crucial that the staff are well-versed in the software to enable highly efficient planning. The alignment of the reference frame, the marking of the Colibri, and the SS8 camera setup should be planned and tested before the surgery to prevent any interference during tracking and to avoid timeloss during surgery. Additional time can be saved by practicing the placement of the reference frame to avoid unstable placement, as this may necessitate restarting the process from the beginning. In general, neuronavigation should be repeatedly tested and practiced on cadavers before being applied to clinical cases.

The time for preoperative positioning was not measured; however, it was noted that the FC group required more complex positioning, as correct lateral positioning is crucial for the success of the procedure. Computer-navigated drilling can be performed with an oblique positioning as well, but lateral positioning facilitates planning of the drill corridor in the authors opinion.

The main limitation of this study is certainly its cadaveric nature with induced sacroiliac luxation and without the typically concurrent soft tissue injuries. The normally concomitant pelvic fractures were simulated by osteotomy of the pubic symphysis. Additionally, both sacroiliac joints were luxated prior to the study, necessitating bilateral surgical approaches, to standardize conditions for all procedures. This resulted in a highly unstable environment conducive to easy mobilization but not representative of the clinical scenario in live animals, where manipulation of the sacral wing can be quite different, especially in cases where the trauma happened several days before surgery and muscular contraction is evident. Furthermore, the study focused solely on the isolated step of drilling into the sacral body, while the subsequent procedural steps were initially disregarded. Although we have attempted to mimic concurrent injuries, the complexity of surrounding soft tissue changes and additional fractures cannot be fully replicated in a live patient. Live animal studies are required to evaluate neuronavigation in clinical conditions. Ideally, a clinical study on cats with unilateral sacroiliac luxation would assess the safety of this technique *in vivo*. In cases of unilateral sacroiliac luxation, additional options for securing the reference frame are available, such as fixation on the non-luxated iliac wing, and the risk of penetrating the spinal canal could be eliminated.

In addition, preoperative and postoperative CBCT images were manually superimposed for accuracy assessment using SS software to compare the final drilling hole to the planned hole. Even as this method allows for direct comparison, it is important to note that manual superimposition can introduce sources of error. The automatic superimposition tool in the SS software proved unreliable due to discrepancies in the bony structures between preoperative and postoperative CT images. These variations were caused by manipulation of the iliac wing during surgery.

A significant drawback for clinical application of neuronavigation is the substantial financial investment required to implement this technology. In addition to the Stealth Station, a compatible CBCT unit (O-arm) is necessary. The more clinical indications are evaluated and described for the use of neuronavigation, the more economically viable such an investment becomes.

## 5 Conclusion

Neuronavigation can be effectively implemented after several practice cases, allowing surgical fixation of sacroiliac luxation in cats. Placement of the reference frame pin must be practiced beforehand to ensure proper positioning. With proper training, it provides real-time feedback and allows performing complex procedures with high accuracy. The new technique shows great potential, and its successful application in clinical cases is conceivable, but must be evaluated in further studies. The required equipment represents a significant financial investment and necessitates a well-coordinated team for successful implementation.

## Data Availability

The raw data supporting the conclusions of this article will be made available by the authors, without undue reservation.
